# Identification of a transcription factor network regulating anti-TNF mediated *IL10* expression in human CD4+ T cells

**DOI:** 10.1093/discim/kyae013

**Published:** 2024-07-27

**Authors:** Giovanni A M Povoleri, Michael L Ridley, Rebecca J Marrow, Sylvine Lalnunhlimi, Sarah E Ryan, Audrey Kelly, Paul Lavender, Leonie S Taams

**Affiliations:** Centre for Inflammation Biology and Cancer Immunology (CIBCI), Department of Inflammation Biology, School of Immunology & Microbial Sciences, King’s College London, London, UK; Centre for Inflammation Biology and Cancer Immunology (CIBCI), Department of Inflammation Biology, School of Immunology & Microbial Sciences, King’s College London, London, UK; Centre for Inflammation Biology and Cancer Immunology (CIBCI), Department of Inflammation Biology, School of Immunology & Microbial Sciences, King’s College London, London, UK; Centre for Inflammation Biology and Cancer Immunology (CIBCI), Department of Inflammation Biology, School of Immunology & Microbial Sciences, King’s College London, London, UK; Centre for Inflammation Biology and Cancer Immunology (CIBCI), Department of Inflammation Biology, School of Immunology & Microbial Sciences, King’s College London, London, UK; King’s Centre for Lung Health, Peter Gorer Department of Immunobiology, School of Immunology & Microbial Sciences, King’s College London, London, UK; King’s Centre for Lung Health, Peter Gorer Department of Immunobiology, School of Immunology & Microbial Sciences, King’s College London, London, UK; Centre for Inflammation Biology and Cancer Immunology (CIBCI), Department of Inflammation Biology, School of Immunology & Microbial Sciences, King’s College London, London, UK

**Keywords:** TNF inhibitor, adalimumab, interleukin-10, CD4+ T cells, RNA-seq, ATAC-seq

## Abstract

CD4+ T cells are key players in immune-mediated inflammatory diseases (IMIDs) through the production of inflammatory mediators including tumour necrosis factor (TNF). Anti-TNF therapy has revolutionized the treatment of several IMIDs and we previously demonstrated that *in vitro* treatment of human CD4+ T cells with anti-TNF promotes anti-inflammatory IL-10 expression in multiple subpopulations of CD4+ T cells. Here we investigated the transcriptional mechanisms underlying the IL-10 induction by TNF-blockade in CD4+ T cells, isolated from PBMCs of healthy volunteers, stimulated *in vitro* for 3 days with anti-CD3/CD28 mAb in the absence or presence of anti-TNF. After culture, CD45RA+ cells were depleted before performing gene expression profiling and chromatin accessibility analysis. Gene expression analysis of CD45RA-CD4+ T cells showed a distinct anti-TNF specific gene signature of 183 genes (*q*-value < 0.05). Pathway enrichment analysis of differentially expressed genes revealed multiple pathways related to cytokine signalling and regulation of cytokine production; in particular, *IL10* was the most upregulated gene by anti-TNF, while the proinflammatory cytokines and chemokines *IFNG*, *IL9*, *IL22*, and *CXCL10* were significantly downregulated (*q*-value < 0.05). Transcription factor motif analysis at the differentially open chromatin regions, after anti-TNF treatment, revealed 58 transcription factor motifs enriched at the *IL10* locus. We identified seven transcription factor candidates for the anti-TNF mediated regulation of IL-10, which were either differentially expressed or whose locus was differentially accessible upon anti-TNF treatment. Correlation analysis between the expression of these transcription factors and *IL10* suggests a role for *MAF*, *PRDM1*, and/or *EOMES* in regulating *IL10* expression in CD4+ T cells upon anti-TNF treatment.

## Graphical Abstract

**Graphical Abstract:**
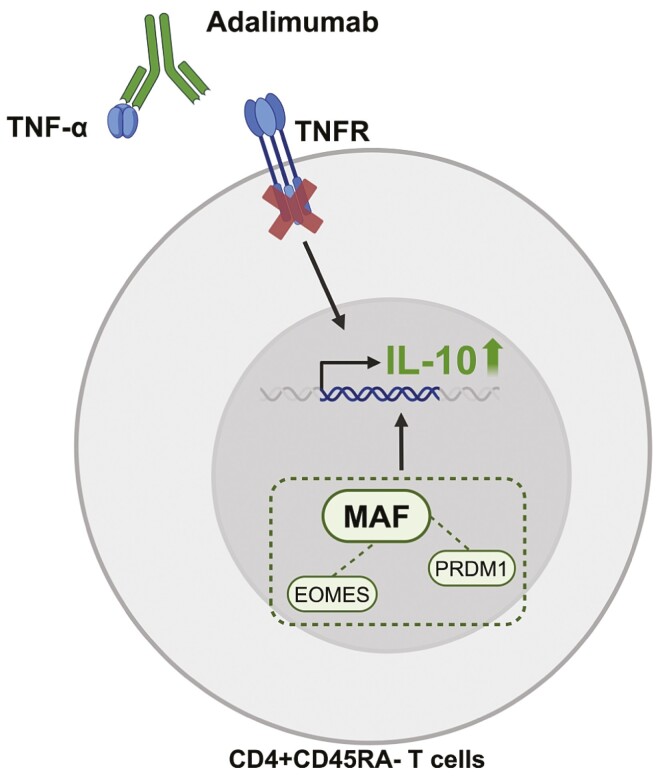

## Introduction

Immune-mediated inflammatory diseases (IMIDs) is a term used to represent a clinically diverse group of diseases that can affect multiple tissues including bone and joint (rheumatoid arthritis and spondyloarthritis), skin (psoriasis and atopic dermatitis), bowel (inflammatory bowel disease), lung (asthma), and central nervous system (multiple sclerosis). These conditions are currently incurable, reduce quality of life and are associated with increased mortality.

CD4+ T cells play a key role in the initiation and/or perpetuation of chronic inflammatory disorders, with different CD4+ T cell subsets being pathogenic in several disease settings. Activated CD4+ T cells contribute to the pathogenesis of IMIDs via effector functions including activation of APCs [1], influencing Ig class switching [2] and by producing multiple inflammatory cytokines including IFNγ, IL-17, and tumour necrosis factor (TNF) [3, 4].

Targeting pathogenic disease mechanisms via cytokine blockade has revolutionized the treatment of IMIDs, particularly following the pioneering application of TNF inhibitors in the treatment of rheumatoid arthritis (RA) [5]. The application of TNF blockade is now a standard of care for several IMIDs including Crohn’s disease, psoriasis and spondyloarthritis [5]. Currently, there are multiple biologics that inhibit or modulate the effects of TNF [6], with adalimumab (ADA), a fully human anti-TNF monoclonal antibody, being one of them.

Anti-TNF biologics can neutralize membrane-bound and soluble TNF, although to different extents; some inhibitors can also induce reverse signalling via membrane-bound TNF. Furthermore, the Fc-regions of anti-TNF mAbs can mediate antibody-dependent cellular cytotoxicity and complement-dependent cytotoxicity [6] or promote direct interaction between monocytes and CD4+ T cells [7]. Thus, TNF inhibitors can act on multiple levels by exerting distinct biological effects on immune cells.

In addition, a connection between anti-TNF treatment and IL-10 production has been proposed in multiple IMIDs [8]: patients with juvenile idiopathic arthritis [9], psoriasis [10], RA [11], or Crohn’s disease [12] were shown to have higher serum or cellular levels of IL-10 after treatment with anti-TNF. The source of IL-10 upon TNF blockade is not always clear in these studies as several immune cell types from the innate (macrophages and dendritic cells) or adaptive (T cells) systems produce IL-10 [13]. Indeed, anti-TNF has been shown to modulate innate immune cell function [14] and induce a macrophage regulatory phenotype characterized by an increased production of IL-10 [15]. TNF blockade can also affect the interaction between innate cells and T cells, leading to the expansion of suppressive Tregs [7] or priming CD4+ T cells for IL-10 production [8, 16]. *In vitro* induction or maintenance of IL-10 production in CD4+ T cells upon TNF inhibition does not require the presence of naïve CD4+ T cells [16] and can also be independent of APC interaction as we have shown previously [16–18].

IL-10 production by most human CD4+ T cell subsets requires ERK activation [19], which suggests a common molecular mechanism within the different subsets; however, *IL10* expression also depends on binding of other transcription factor including the transcription factors specific protein 1 [20], SP3 [21], CCAAT/enhancer binding protein-β (C/EBPβ) [22], IFN-regulatory factor 1, and STAT3 [23]. The list of genes involved in the regulation of IL-10 in human CD4+ T cells is expanding, with MAF suggested as a universal transcription factor for the regulation of IL-10 production [19, 24–26]. MAF can act synergistically or interact with other transcription factors including aryl hydrocarbon recepto [27] and PR domain zinc finger protein 1 (BLIMP-1) [28] leading to a positive regulation of IL-10 production. BLIMP-1, which is encoded by the *PRDM1* gene, has also been shown to positively regulate IL-10 production in CD4+ T cells independently of MAF [29–31]. Regulation of IL-10 production by CD4+ T cells is thus not linear and relies on the interplay of transcription factors.

The aim of this study was to further elucidate the molecular mechanism by which anti-TNF regulates IL-10 expression in CD4+ T cells. We investigated the transcriptomic and chromatin changes in human CD4+ T cells following treatment with anti-TNF mAb. This led to the identification of a transcription factor module consisting of *MAF*, *PRDM1*, and *EOMES*, which may underlie the modulation of IL-10 expression by TNF inhibition.

## Materials and methods

### Cell isolation

Peripheral blood samples were obtained from healthy adult volunteers. Peripheral blood mononuclear cells were isolated by density gradient centrifugation using Lymphoprep (Axis-Shield, Oslo, Norway). CD4+ T cells were isolated by magnetic-activated cell sorting (MACS) using the CD4+ T cell Isolation Kit II (Miltenyi Biotec, Bergisch-Gladbach, Germany). Average purities were 98% for CD4+ T cells. After 3 days of culture, CD4+ T cells were depleted of CD45RA+ cells using CD45RA MicroBeads (Miltenyi Biotec). The study was approved by the Bromley Research Ethics Committee (06/Q0705/20), and written informed consent was obtained from all participants.

### CD4+ T cell culture

Cells were cultured at 37°C with 5% CO_2_ in RPMI 1640 medium (Gibco) supplemented with 10% heat-inactivated fetal bovine serum (Sigma) and 1% penicillin, streptomycin and l-glutamine (all from Gibco). MACS-isolated CD4+ T cells were stimulated at 10^6^/ml with 1.25 µg/ml of plate-bound anti-CD3 (clone OKT3; BD Biosciences) and 1 µg/ml of soluble anti-CD28 (clone CD8.2; BD Biosciences) in the absence or presence of 1 µg/ml of adalimumab (ADA, Abbott Laboratories, Chicago, USA) for 3 days.

### Flow cytometry

To assess intracellular cytokine expression before and after cell culture in the presence or absence of adalimumab, cells were stimulated for 3 h in the presence of phorbol 12-myristate 13-acetate (PMA; 50 ng/ml, Sigma–Aldrich), ionomycin (750 ng/ml, Sigma–Aldrich), and GolgiStop (BD Biosciences). Cells were labelled with a fixable viability dye (LIVE/DEAD fixable dead cell stains, ThermoFisher Scientific), washed and stained extracellularly with CD4 Pacific Blue (SK3; BioLegend), CD45RA FITC (HI100; BioLegend) and CD45RO APC (UCHL1; BioLegend). Cells were then fixed in 2% PFA (paraformaldehyde, Sigma–Aldrich) and permeabilized with 0.5% saponin (Thermo Fisher Scientific). Cells were stained intracellularly with IL-10 PE (JES3-9D7; BioLegend). Stained cells were acquired using a FACSCantoII (BD Biosciences); in most experiments, 100 000 T cell events were recorded. All flow cytometry data were analysed using FlowJo software (version 10, Tree Star, Inc., Ashland, USA).

### RNA-seq

RNA was isolated from 500 000 CD4+ CD45RA– cells after 3 days of stimulation in the presence or absence of 1 ug/ml adalimumab. Total RNA (RNA integrity number > 9) was used to construct RNA sequencing libraries. Starting with 100 ng total RNA, cDNA synthesis and amplification were performed using a NEBNext Ultra II Directional RNA Library Prep Kit for Illumina (NEB, MA, USA) following the manufacturer’s protocol. Dual-indexed libraries were prepared using the NEBNext^®^ Multiplex Oligos for Illumina^®^ (Dual Index Primers Set 1) following the manufacturer’s protocol. The libraries were sequenced at Genewiz Ltd (Genewiz, Germany) on an Illumina NovaSeq 2 × 150 bp at an average of 35 M reads per sample. The reads were mapped against the genome using STAR on default parameters against the human hg38 reference genome release 32. Differential gene expression analysis (*q*-value < 0.05) was performed using DESeq2. Functional enrichment analysis of differential gene lists to identify significantly enriched biological functions and pathways was performed using gprofiler [32] using standard settings. Raw data can be accessed at GSE216688.

### ATAC-seq

Nuclei from 50 000 CD4+ CD45RA– cells stimulated for 3 days in the presence or absence of ADA were isolated and ATAC-seq was performed according to a published protocol [33]. Paired-end libraries (50 cycles) were prepared according to the ATAC-seq protocol (see above). To obtain the open chromatin regions, reads were aligned to hg38 using Bowtie v2.3.42 with parameters [--maxins 175 --no-discordant --no-mixed]. Properly paired and uniquely mapped alignments were extracted using bamFilter v2.4.1 with parameters[isMapped = true, isPaired = true, isDuplicate = false, reference=!chrM]. The open chromatin regions were identified using the findPeaks tool from Homer v4.11 [34] with parameters [-style factor -size 300 -minDist 50 -fdr 0.001 -norm 40000000 -tbp 0 -region -o]. For each donor, differential open chromatin regions, comparing No ADA to ADA treated samples, were extracted using Homer getDifferentialPeaks with parameter –*F* 1.5. Differential open chromatin regions from all donors were merged using Homer mergePeaks with parameter -*d* 100. To identify motifs that are enriched at differential open chromatin regions in each donor, we used Homer findMotifsGenome.pl on all known motifs with parameter [-size given]. Raw data can be accessed at GSE216688.

### Statistical analysis

Statistical testing was performed with GraphPad Prism 9.0 (GraphPad, San Diego, CA, USA). Data sets were tested using the appropriate non-parametric test as indicated in figure legends. The correlation matrix between the expression of *IL10* and the seven transcription factor candidates reports the two-tailed Spearman r correlation computed for every combination. *P*-values or *q*-values (*P*-values corrected for multiple testing, as specified in figure legends) < 0.05 were considered statistically significant.

## Results

### *In vitro* treatment with anti-TNF confers a distinct gene signature onto memory CD4+ T cells characterized by differential expression of multiple cytokines

**Figure 1:**
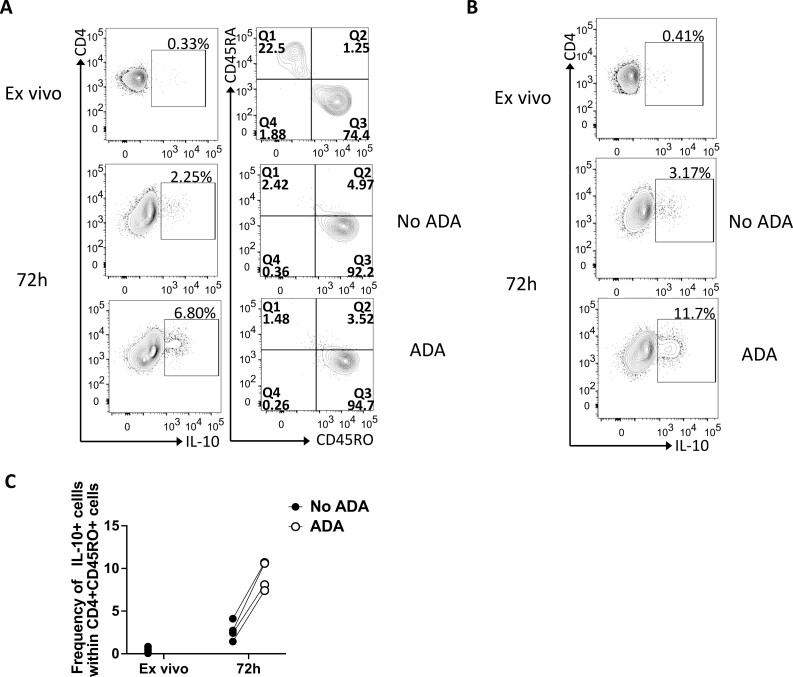
IL-10 production is restricted to memory T cells and significantly increased by anti-TNF at 72 h. (**A**) Representative flow cytometry plot showing frequencies of IL‐10+ cells among CD4+ T cells (left) and frequencies of CD45RA+ and CD45RO+ cells within CD4+ IL-10+ cells (right) *ex vivo* or 72 h post stimulation with aCD3/CD28 mAb, in the absence or presence of ADA. (**B**) Representative flow cytometry plot showing frequencies of IL‐10 producing cells in CD4+ CD45RA– T cells *ex-vivo* (*ex vivo*) or 72 h post stimulation with aCD3/CD28 mAb, in the absence or presence of adalimumab (ADA). (**C**) Cumulative plots showing the frequencies of populations from (B) *ex vivo* or 72 h post stimulation with aCD3/CD28 mAb, in the absence (filled symbol) or presence (open symbol) of ADA in the cells that were taken forward for RNAseq and ATACseq (Wilcoxon matched-pairs signed rank test, *n* = 4).

We previously demonstrated that *in vitro* anti-TNF treatment led to a significant increase in IL-10 production 72 h post stimulation both at RNA and protein levels [11, 16–18]. To elucidate the molecular mechanism behind the anti-TNF-mediated induction of IL-10, we performed combined RNA-seq and ATAC-seq. We focused on memory CD4+ T cells as we identified that IL-10 production is restricted to that population (Fig. 1A). CD4+ T cells were stimulated with anti-CD3 and anti-CD28 (aCD3/CD28) mAb for 72 h in the absence or presence of 1 μg/ml of the anti-TNF drug adalimumab (ADA), without PMA and ionomycin restimulation. We then enriched for memory CD4+ T cells by depleting CD45RA+ cells, followed by isolation of RNA and nuclei to perform paired RNA and ATAC-sequencing (Supplementary Fig. S1). We confirmed that there was an anti-TNF-mediated increase in IL-10 production (using PMA and ionomycin restimulation) by the CD4+ CD45RA– T cells, defined from now on as memory T cells, which we used as input for sequencing (Fig. 1B and C).

**Figure 2:**
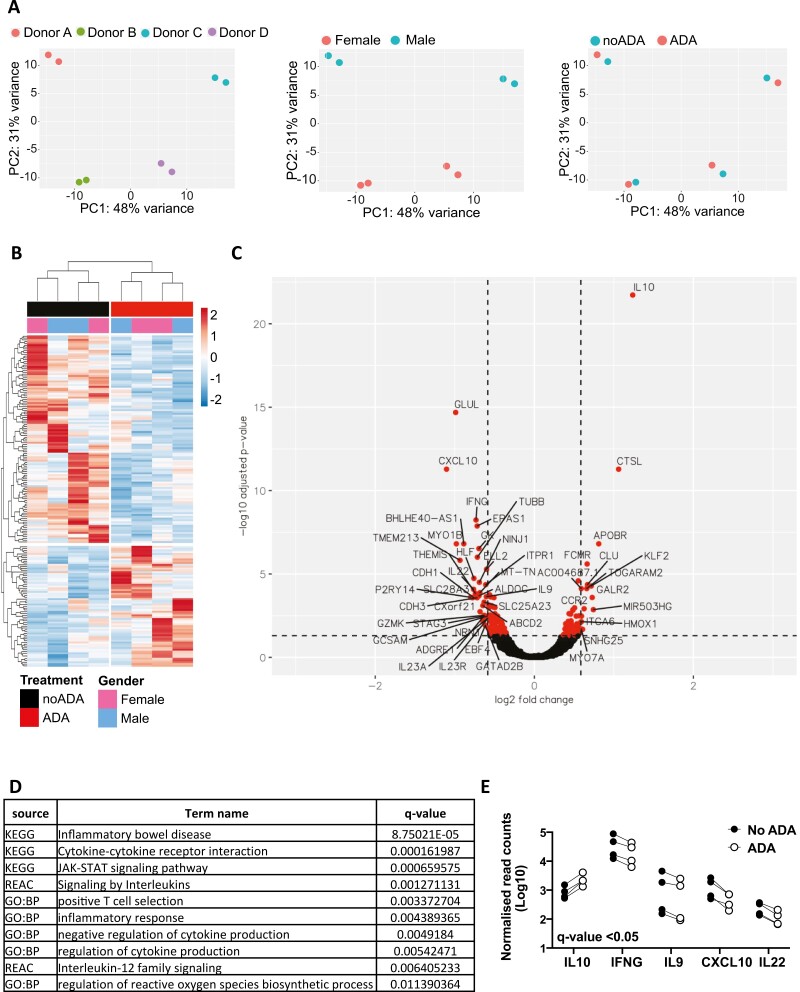
anti-TNF treatment confers a specific gene signature characterized by increased *IL10* and decreased inflammatory cytokine genes. (**A**) PCA plots of gene expression showing clustering of samples based on donor, gender and anti-TNF treatment. (**B**) Heatmap of differentially expressed (DE) genes between ADA and no ADA-treated cells at 72 h post stimulation (*q*-value < 0.05). (**C**) Volcano plot showing differentially expressed genes (coloured symbols) between no ADA and ADA-treated cells at 72 h. Threshold lines at *q*-value < 0.05 and +1.5 and –1.5-fold change. (**D**) Top 10 significant biological functions and pathways (*q*-value < 0.05) from functional enrichment analysis of DE genes with a 1.5-fold change cutoff. (**E**) Cumulative plot showing normalized read counts of DE cytokine and chemokine genes at 72 h post stimulation with aCD3/CD28 mAb, in the absence (filled symbol) or presence (open symbol) of ADA.

Principal component analysis (PCA) of the RNA-seq data showed that donor and gender were the dominant sources of variation in the dataset, with anti-TNF treatment having a relatively subtle effect (Fig. 2A). Differential gene expression analysis revealed that anti-TNF treatment conferred a unique gene signature in memory CD4+ T cells, with 67 upregulated and 115 downregulated genes (*q*-value < 0.05). Notably, *IL10* was found to be the top upregulated gene by adalimumab (Fig. 2B and C and Table 1). Functional enrichment analysis of the differentially expressed genes revealed enrichment for multiple gene sets associated with cytokine signalling and regulation of cytokine production pathways (Fig. 2D). In particular, we found that CD4+ T cells that were stimulated in the presence of anti-TNF for 72 h showed a significant decrease in the expression of the inflammatory cytokines and chemokines *IFNG*, *IL9*, *IL22*, and *CXCL10*, in parallel to the significant increase in *IL10* expression (Fig. 2E). Analysis of gene changes at 24 h post anti-TNF treatment showed a similar significant decrease in the inflammatory cytokine and chemokine genes *IFNG*, *IL9*, *IL22*, and *CXCL10*, as well as in *IL17F* and *IL5*, with an increase in *IL2*, but not *IL10* expression ( Supplementary Fig. S2A and B), consistent with our previous data that showed that the differential expression of IL-10 became manifest at later timepoints only [16, 18]. Of interest, we found that *BHLHE40*, a *MAF* repressor that when deficient in CD4 Th1 cells leads to decreased IFN-γ and increased IL-10 [35], as well as its antisense transcript *BHLHE40-AS1,* which has been suggested as a modulator of a proinflammatory cytokine signature [36], were significantly downregulated at 72 h, but not 24 h, post anti-TNF treatment ( Supplementary Fig. S2C). Taken together, these data indicate that *in vitro* anti-TNF treatment alters the cytokine gene profile of memory CD4+ T cells leading to a downregulation of inflammatory cytokine gene expression at both 24 h and 72 h, and a differential upregulation of immunomodulatory *IL10* at 72 h.

**Table 1:** list of differentially expressed (DE) genes between no ADA and ADA-treated cells at 72 h post stimulation (*q*-value < 0.05)

IL10	RPL36A	SPN	PPP2R5D	GNG4	GATAD2B
CTSL	MINOS1	SPN	IL12RB2	CCDC6	GZMK
APOBR	RBM25	ENGASE	PIK3C2B	RRN3	SLC25A23
MIR503HG	YJEFN3	GATD1	EXT1	PPFIBP1	STAG3
GALR2	NPDC1	SNHG3	BHLHE40	GNA15	NRN1
KLF2	LUC7L	FAM107B	SLAIN1	CCNK	ABCD2
CLU	AL390728.6	RAPGEF1	NFE2L3	ZMIZ1	ADGRE1
FCMR	IL7R	TANK	AHCYL2	STARD10	CXorf21
TOGARAM2	AC010761.1	PPP1R16B	EPB41L2	ARHGAP31	MT-TN
CCR2	ELMO3	CXCR4	TUBA1B	SH3TC1	NINJ1
SNHG25	SDF2L1	PFKFB3	CAP1	DDIT4	ALDOC
ITGA6	DUSP6	SHC1	PPP2R5B	MVB12B	GK
HMOX1	GPAT3	PRPF8	CLIC4	SEC24C	TUBB
MYO7A	TMEM2	FBRS	HDGFL3	BNIP3L	EPAS1
AC004687.1	CRACR2B	CRTAP	COL9A2	LINC01215	CDH3
EGFL6	CSF2RB	YWHAE	BCAT1	CPNE5	IFNG
EPHB4	APOL1	MED12	UNC119	RGS1	THEMIS
HAGHL	PGGHG	CCNG1	TIMD4	FAM60A	HLF
SLC14A1	SNHG12	ELK1	LSS	ABCC1	IL22
ZBP1	RALGDS	TNIP1	FOPNL	TNFAIP3	CDH1
IKZF3	JAML	MALT1	IZUMO4	SMAP2	SLC28A3
SNORD104	MALAT1	TNFSF10	MCM2	FTH1	P2RY14
IFI44L	GZMH	FBXL14	IQCG	RFFL	BHLHE40-AS1
IFI27	LTB4R	NCOA7	RIPOR1	FEZ1	TMEM213
NEAT1	LUC7L3	EGLN1	SNX10	IL23R	MYO1B
HBD	C1orf228	CD74	GPR160	IL23A	GLUL
BST2	RASGRP2	CNOT1	FOXP3	GCSAM	CXCL10
AC245060.5	XBP1	NFKB2	DENND5A	ELL2	
FAM173A	CCR5	CHST2	MLLT6	IL9	
PLEKHN1	MAF	TIFA	BIRC3	ITPR1	
CPNE7	CTLA4	FOXP4	SGPP2	EBF4	

### Anti-TNF treatment changes chromatin accessibility revealing a putative transcription factor network controlling IL-10 expression

**Figure 3:**
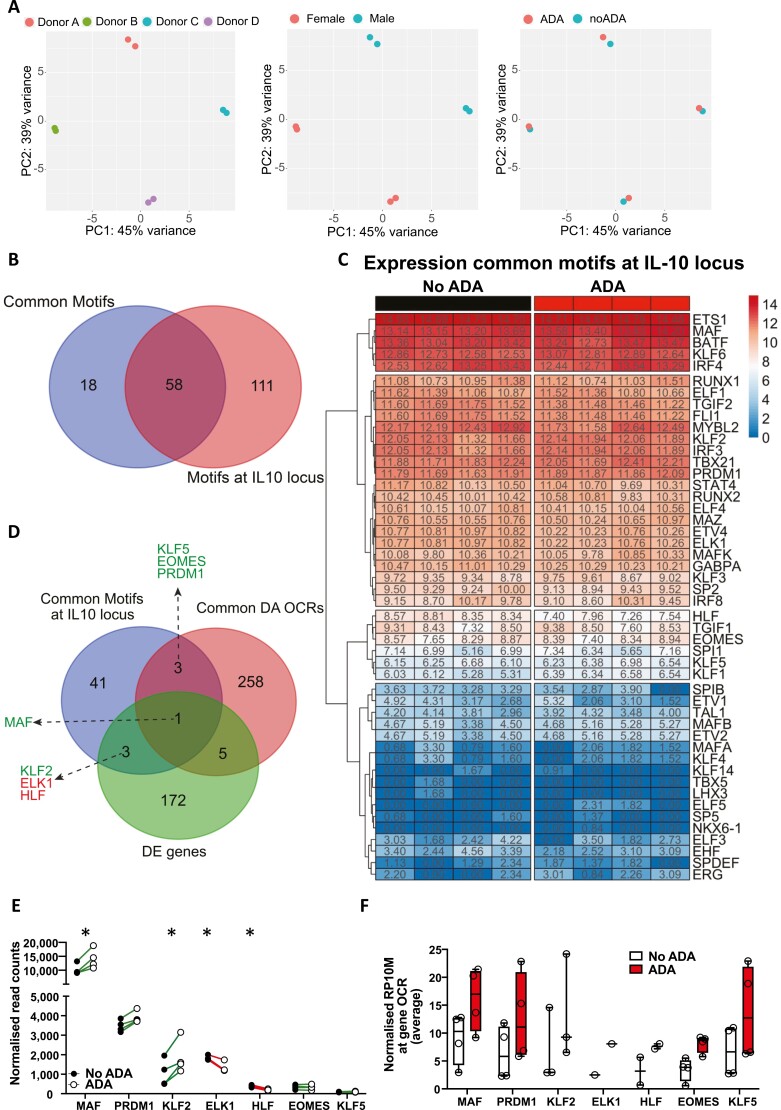
anti-TNF treatment modulates chromatin accessibility and reveals a putative transcription factor network regulating *IL10*. (**A**) PCA plots of chromatin accessibility showing clustering of samples based on donor, gender, and anti-TNF treatment. (**B**) Venn diagram showing the overlap of common transcription factor motifs from Fig. S3B significantly enriched (*P* < 0.05) in ADA-treated cells at 72 h post stimulation with transcription factor motifs mapping at the *IL10* locus. (**C**) Heatmap showing normalized read counts (log2 transformed) of the common motifs mapped at the *IL10* locus (intersection from B) in CD45RA- T cells 72 h post stimulation with aCD3/CD28 mAb, in the absence (left panel) or presence (right panel) of ADA. (**D**) Venn diagram showing the overlap of common transcription factor motifs significantly enriched and mapping at the *IL10* locus, the common differentially accessible (DA) OCRs and differentially expressed (DE) genes from ADA-treated cells at 72 h post stimulation. (**E**) Cumulative plot showing normalized read counts of highlighted genes in D at 72 h post stimulation with aCD3/CD28 mAb, in the absence (filled symbol) or presence (open symbol) of ADA. (**F**) Cumulative plot showing from each donor (round symbols) averaged normalized peak intensities (‘tags’) per 10 million reads (RP10M) for differential OCRs at the gene loci from D 72 h post stimulation with aCD3/CD28 mAb, in the absence or presence of ADA.

To identify how anti-TNF treatment mechanistically alters the regulome of CD4+ T cells leading to increased IL-10 expression at 72 h, we performed Assay for Transposase-Accessible Chromatin using sequencing (ATAC-seq) on the same samples. We identified 152 595 peaks (i.e. regions of accessible chromatin) within our samples. PCA of accessible chromatin regions shared between donors showed a similar result to our RNA-seq analysis with donor and gender being the largest sources of variation in the dataset, followed by anti-TNF treatment (Fig. 3A). Despite this variability, we identified 2,113 differential peaks (Poisson *P*-value < 0.0001 and fold change ≥ 1.5) in anti-TNF treated samples compared to no drug control (heatmap shown in  Supplementary Fig. S3A).

We performed motif enrichment analysis (*P*-value < 0.05) of known transcription factors at differentially accessible (DA) open chromatin regions (OCRs) in anti-TNF-treated cells at 72 h post stimulation in each donor independently. This analysis identified 76 enriched motifs upon anti-TNF treatment that were shared by all donors (Supplementary Fig. S3B and Table 2). Since our interest was to determine which transcription factors may regulate IL-10 production upon anti-TNF treatment, we focussed our analysis on the shared transcription factor motifs that mapped to the *IL10* locus, which reduced our candidate pool down to 58 transcription factors and fusion proteins (included by the motif finding software available from ChIP-seq data) (Fig. 3B and Table 3). We further refined our candidate list by focussing on the common transcription factors mapping at the *IL10* locus whose expression we could measure in our RNA-seq data, which therefore excluded fusion proteins (Fig. 3C). Finally, to identify potential anti-TNF dependent IL-10 regulators, we assessed which common transcription factors binding at the *IL10* locus were either differentially expressed (DE genes) or had their gene locus differentially accessible (DA OCRs) after anti-TNF treatment (Fig. 3D). This analysis revealed seven transcription factors mapping to the *IL10* locus that were either differentially expressed (upregulated or downregulated) and/or differentially accessible upon treatment with anti-TNF. Of these seven, the transcription factor MAF, which was shown previously to be a transcriptional regulator of *IL10*, was both differentially upregulated and differentially accessible upon anti-TNF treatment. In addition, *KLF5*, *EOMES* and *PRDM1* were found to be differentially accessible upon ADA treatment, whilst *KLF2* was differentially upregulated (Fig. 3E and F). We also identified *ELK1* and *HLF* as being differentially downregulated after anti-TNF treatment, suggesting a potential inhibitory role (Fig. 3E). When we evaluated these transcription factors at 24 h we found only *KLF2* differentially upregulated after anti-TNF, however, both loci for *MAF* and *PRDM1* were differentially accessible in all donors ( Supplementary Fig. S3C and D).

**Table 2:** list of common TFs motifs significantly enriched (*P* < 0.05) at differentially accessible OCRs in ADA-treated cells at 72 h post stimulation

ELF1(ETS)/Jurkat-ELF1-ChIP-Seq(SRA014231)/Homer	ETS(ETS)/Promoter/Homer
Etv2(ETS)/ES-ER71-ChIP-Seq(GSE59402)/Homer	EHF(ETS)/LoVo-EHF-ChIP-Seq(GSE49402)/Homer
IRF4(IRF)/GM12878-IRF4-ChIP-Seq(GSE32465)/Homer	RUNX-AML(Runt)/CD4+-PolII-ChIP-Seq(Barski_et_al.)/Homer
ETS:RUNX(ETS,Runt)/Jurkat-RUNX1-ChIP-Seq(GSE17954)/Homer	EWS:FLI1-fusion(ETS)/SK_N_MC-EWS:FLI1-ChIP-Seq(SRA014231)/Homer
BMYB(HTH)/Hela-BMYB-ChIP-Seq(GSE27030)/Homer	Sp2(Zf)/HEK293-Sp2.eGFP-ChIP-Seq(Encode)/Homer
KLF3(Zf)/MEF-Klf3-ChIP-Seq(GSE44748)/Homer	MAF_MA1520.1
Fli1(ETS)/CD8-FLI-ChIP-Seq(GSE20898)/Homer	IRF2(IRF)/Erythroblas-IRF2-ChIP-Seq(GSE36985)/Homer
Ets1-distal(ETS)/CD4+-PolII-ChIP-Seq(Barski_et_al.)/Homer	SPDEF(ETS)/VCaP-SPDEF-ChIP-Seq(SRA014231)/Homer
Egr1(Zf)/K562-Egr1-ChIP-Seq(GSE32465)/Homer	ETS1(ETS)/Jurkat-ETS1-ChIP-Seq(GSE17954)/Homer
Bach2(bZIP)/OCILy7-Bach2-ChIP-Seq(GSE44420)/Homer	Elk4(ETS)/Hela-Elk4-ChIP-Seq(GSE31477)/Homer
ERG(ETS)/VCaP-ERG-ChIP-Seq(GSE14097)/Homer	Elk1(ETS)/Hela-Elk1-ChIP-Seq(GSE31477)/Homer
ISRE(IRF)/ThioMac-LPS-Expression(GSE23622)/Homer	KLF6(Zf)/PDAC-KLF6-ChIP-Seq(GSE64557)/Homer
MafF(bZIP)/HepG2-MafF-ChIP-Seq(GSE31477)/Homer	PU.1:IRF8(ETS:IRF)/pDC-Irf8-ChIP-Seq(GSE66899)/Homer
PU.1-IRF(ETS:IRF)/Bcell-PU.1-ChIP-Seq(GSE21512)/Homer	EWS:ERG-fusion(ETS)/CADO_ES1-EWS:ERG-ChIP-Seq(SRA014231)/Homer
ELF3(ETS)/PDAC-ELF3-ChIP-Seq(GSE64557)/Homer	Tgif1(Homeobox)/mES-Tgif1-ChIP-Seq(GSE55404)/Homer
MafB(bZIP)/BMM-Mafb-ChIP-Seq(GSE75722)/Homer	ELF5(ETS)/T47D-ELF5-ChIP-Seq(GSE30407)/Homer
IRF:BATF(IRF:bZIP)/pDC-Irf8-ChIP-Seq(GSE66899)/Homer	Tbx5(T-box)/HL1-Tbx5.biotin-ChIP-Seq(GSE21529)/Homer
Nkx6.1(Homeobox)/Islet-Nkx6.1-ChIP-Seq(GSE40975)/Homer	Jun-AP1(bZIP)/K562-cJun-ChIP-Seq(GSE31477)/Homer
KLF14(Zf)/HEK293-KLF14.GFP-ChIP-Seq(GSE58341)/Homer	bZIP:IRF(bZIP,IRF)/Th17-BatF-ChIP-Seq(GSE39756)/Homer
BORIS(Zf)/K562-CTCFL-ChIP-Seq(GSE32465)/Homer	Eomes(T-box)/H9-Eomes-ChIP-Seq(GSE26097)/Homer
Bach1(bZIP)/K562-Bach1-ChIP-Seq(GSE31477)/Homer	ETV4(ETS)/HepG2-ETV4-ChIP-Seq(ENCODE)/Homer
CTCF(Zf)/CD4+-CTCF-ChIP-Seq(Barski_et_al.)/Homer	Tgif2(Homeobox)/mES-Tgif2-ChIP-Seq(GSE55404)/Homer
MafA(bZIP)/Islet-MafA-ChIP-Seq(GSE30298)/Homer	Pdx1(Homeobox)/Islet-Pdx1-ChIP-Seq(SRA008281)/Homer
Maz(Zf)/HepG2-Maz-ChIP-Seq(GSE31477)/Homer	PRDM1(Zf)/Hela-PRDM1-ChIP-Seq(GSE31477)/Homer
HLF(bZIP)/HSC-HLF.Flag-ChIP-Seq(GSE69817)/Homer	Tbet(T-box)/CD8-Tbet-ChIP-Seq(GSE33802)/Homer
Nrf2(bZIP)/Lymphoblast-Nrf2-ChIP-Seq(GSE37589)/Homer	RUNX(Runt)/HPC7-Runx1-ChIP-Seq(GSE22178)/Homer
Elf4(ETS)/BMDM-Elf4-ChIP-Seq(GSE88699)/Homer	NF-E2(bZIP)/K562-NFE2-ChIP-Seq(GSE31477)/Homer
KLF5(Zf)/LoVo-KLF5-ChIP-Seq(GSE49402)/Homer	STAT4(Stat)/CD4-Stat4-ChIP-Seq(GSE22104)/Homer
IRF8(IRF)/BMDM-IRF8-ChIP-Seq(GSE77884)/Homer	KLF1(Zf)/HUDEP2-KLF1-CutnRun(GSE136251)/Homer
IRF3(IRF)/BMDM-Irf3-ChIP-Seq(GSE67343)/Homer	NFAT:AP1(RHD,bZIP)/Jurkat-NFATC1-ChIP-Seq(Jolma_et_al.)/Homer
SpiB(ETS)/OCILY3-SPIB-ChIP-Seq(GSE56857)/Homer	MafK(bZIP)/C2C12-MafK-ChIP-Seq(GSE36030)/Homer
NFE2L2(bZIP)/HepG2-NFE2L2-ChIP-Seq(Encode)/Homer	RUNX2(Runt)/PCa-RUNX2-ChIP-Seq(GSE33889)/Homer
ETV1(ETS)/GIST48-ETV1-ChIP-Seq(GSE22441)/Homer	RUNX1(Runt)/Jurkat-RUNX1-ChIP-Seq(GSE29180)/Homer
Klf4(Zf)/mES-Klf4-ChIP-Seq(GSE11431)/Homer	SCL(bHLH)/HPC7-Scl-ChIP-Seq(GSE13511)/Homer
KLF2_MA1515.1	GABPA(ETS)/Jurkat-GABPa-ChIP-Seq(GSE17954)/Homer
Lhx3(Homeobox)/Neuron-Lhx3-ChIP-Seq(GSE31456)/Homer	PU.1(ETS)/ThioMac-PU.1-ChIP-Seq(GSE21512)/Homer
CEBP(bZIP)/ThioMac-CEBPb-ChIP-Seq(GSE21512)/Homer	Sp5(Zf)/mES-Sp5.Flag-ChIP-Seq(GSE72989)/Homer
Egr2(Zf)/Thymocytes-Egr2-ChIP-Seq(GSE34254)/Homer	IRF1(IRF)/PBMC-IRF1-ChIP-Seq(GSE43036)/Homer

**Table 3.** list of common TFs motifs significantly enriched (*P* < 0.05) in treated cells at 72 h post stimulation and mapping at the *IL10* locus

IRF4	EHF	KLF2	Etv2	Maz	ELF1
PU.1	ELF3	bZIP:IRF	RUNX-AML	SpiB	PRDM1
RUNX	KLF5	Sp2	Eomes	ETS	EWS:ERG-fusion
IRF:BATF	ERG	ELF5	MafB	HLF	RUNX2
BMYB	PU.1-IRF	Tbet	MAF	GABPA	Lhx3
ETS:RUNX	MafA	Tbx5	Elk1	RUNX1	Nkx6.1
KLF1	Klf4	Elf4	NFAT:AP1	KLF14	KLF6
Tgif1	STAT4	ETS1	ETV4	EWS:FLI1-fusion	KLF3
Sp5	ETV1	Fli1	Ets1-distal	IRF8	
SPDEF	IRF3	Tgif2	MafK	SCL	

**Figure 4:**
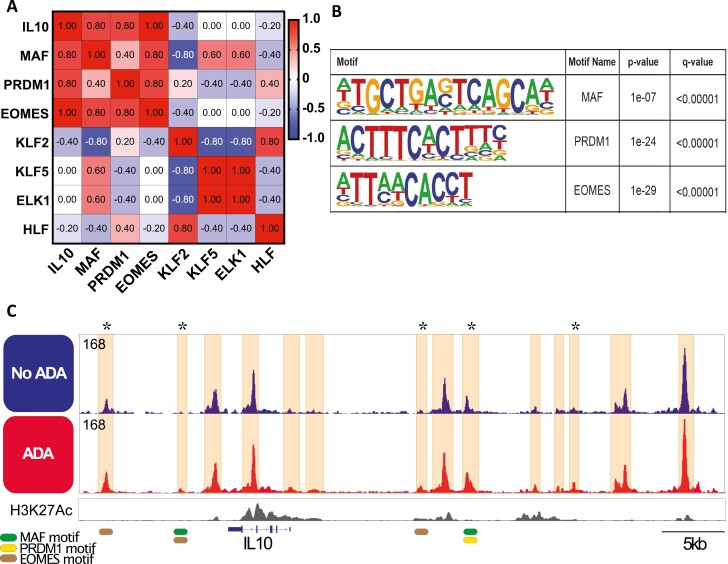
*MAF*, *PRDM1*, and *EOMES* form a putative transcription factor module regulating *IL10* expression upon anti-TNF treatment. (**A**) Heatmap showing Spearman correlations of *IL10* gene expression and transcription factors from Fig. 3E at 72 h posttreatment with anti-TNF. (**B**) DNA binding motif sequence logo and *q*-value (Benjamini) for *MAF*, *PRDM1*, and *EOMES* from motif enrichment analysis at differentially accessible peaks from ATAC-seq of CD4+ CD45RA– T cells 72 h post stimulation. (**C**) Representative example of OCR at the *IL10* locus 72 h post stimulation with aCD3/CD28 mAb, in the absence (top track) or presence (bottom track) of ADA; layered H3K27Ac track (ENCODE) added for reference. OCRs corresponding to ATAC peaks are highlighted. Differential peaks (indicated by asterisks) in ADA treated T cells (Poisson *P*-value = 0.0001 and fold change = 1.5 cutoffs) and mapping of MAF, PRDM1 and EOMES binding motifs enriched at differential peaks are shown.

We next performed correlation and linear regression analysis of gene expression at 72 h posttreatment with anti-TNF and found a highly positive correlation between *MAF*, *PRDM1*, or *EOMES* with *IL10*, as well as a positive correlation between these three transcription factors (Fig. 4A and Supplementary Fig. S4) suggesting potential synergy; in contrast, either no or a negative correlation was found between *KLF2*, *KLF5*, *ELK1*, and *HLF* with *IL10* (Fig. 4A and Supplementary Fig S4). Furthermore, we found that the motifs for AP-1/MAF, PRDM1, and EOMES (Fig. 4B) could bind at multiple OCRs either at distinct or shared sites on the *IL10* locus (Fig. 4C). Taken together, these data show that anti-TNF leads to chromatin remodelling in CD4+ T cells whilst transcription factor motif analysis at the differential OCRs after treatment with anti-TNF indicates a putative transcription factor network that can regulate *IL10* expression.

## Discussion

Since our initial observations that anti-TNF treatment led to an increase in anti-inflammatory IL-10 in T cells from healthy donors and in patients with inflammatory arthritis [11], we have worked towards understanding the molecular mechanism underlying the anti-TNF dependent regulation of IL-10. Our previous work showed that anti-TNF treatment *in vitro* maintained a long-term increased production of IL-10 in multiple T-cell populations [16, 17], which could indicate transcriptional regulation. We initially hypothesized that the transcription factor *IKZF3* (encoding Aiolos), which is upregulated upon anti-TNF treatment [11], had a direct role in regulating the anti-TNF effect on IL-10. Detailed analysis however demonstrated that IKZF3/Aiolos is associated with, but not sufficient to drive IL-10 [18]. Thus, in this report, we sought to uncover the mechanism underlying the maintenance of the IL-10 program in CD4+ T cells by anti-TNF.

Despite a high level of donor and gender variation, we were able to identify anti-TNF-dependent transcriptional changes in bulk memory CD4+ T cells. Reassuringly, the most upregulated gene in our dataset was *IL10*. Furthermore, we found a number of pro-inflammatory cytokines and chemokines significantly downregulated upon anti-TNF treatment. In particular, *IFNG* was the most highly expressed inflammatory cytokine that was significantly downregulated in all donors upon anti-TNF treatment. It should be noted that we previously did not observe such a strong effect of anti-TNF on IFN-γ at the protein level [17]; this may be due to the effect of PMA and ionomycin restimulation in those assays, which has been shown to increase IFN-γ production [37].

In our attempt to gain mechanistic insight into the molecular regulation of *IL10* by anti-TNF, we performed motif discovery analysis at differential OCRs upon treatment with anti-TNF to define a putative transcription factor network. Of our final seven transcription factor candidates we focussed on those that were either differentially regulated, or whose loci were more accessible after anti-TNF and those whose expression positively correlated with *IL10*.

Perhaps unsurprisingly, the prime candidate we found for the anti-TNF-dependent regulation of IL-10 was *MAF*. Multiple studies have demonstrated its correlation with *IL10* regulation either directly or synergistically with other transcription factors, including *PRDM1* [25, 26]. *PRDM1*, which encodes the transcriptional regulator BLIMP-1, has been described itself as a requirement for IL-10 production in multiple cell populations including mucosal Tregs [30], cytotoxic T lymphocytes [38] and IL-10-producing effector Th cells [28].

There is less evidence of EOMES directly regulating IL-10, although it has been described as a lineage-defining transcription factor in human IL-10-producing Tr1-like cells either independently [39] or synergistically with Blimp-1 [40]. Indeed, our own data showed that not only the expression of *MAF*, *PRDM1*, and *EOMES* was positively correlated with *IL10* expression, but also with each other, suggesting that IL-10 regulation could be the result of the synergistic effect of multiple transcription factors. Interestingly, and in line with our findings, a recent *in vitro* study using mouse CD4+ T cells showed that c-Maf and BLIMP-1 not only work synergistically but can also target each other and act as positive modulators of *Il10* gene expression, while at the same time acting as negative regulators of *Ifng* by directly binding to the loci encoding these cytokines in Th1 effector cells upon differentiation with IL-12 and IL-27 [41]. While there are differences between the mouse and human systems, the conserved nature of these transcription factors would suggest a similarity in their transcriptional regulation of cytokine production.

Our data showed gender as a potential source of variation in our transcriptomic and regulome data, upon treatment with anti-TNF. There has been an increased interest in the differences found in immune responses and immune homeostasis between genders, which has been partially correlated with disease susceptibility and response to treatment [42]. This highlights the importance of considering gender when studying immunological responses. It would be interesting and relevant to explore how gender affects the T-cell response to activation, both in the presence or absence of anti-TNF and whether there is a direct effect on IL-10 regulation, production or even a variation in the kinetics of its modulation.

In conclusion, the work presented here reveals changes caused by anti-TNF treatment in CD4+ memory T cells at both transcriptional and regulome levels. Our data support the concept that anti-TNF alters the immune response of memory T cells by negatively modulating inflammatory mediators, while positively modulating anti-inflammatory IL-10. We provide evidence for a putative transcription factor module regulating *IL10* expression upon anti-TNF treatment consisting of *MAF*, which showed the strongest evidence in our data, together with *PRDM1* and *EOMES*. These findings reveal potential targets for novel therapeutic strategies that could aim at stabilizing the immune-modulating effect of anti-TNF treatment on human CD4+ T cells.

## Supplementary data

Supplementary data is available at *Discovery Immunology* online.

kyae013_suppl_Supplementary_Figures_S1-S4

## Data Availability

According to UK research councils’ Common Principles on DataPolicy, all RNA-seq, and ATAC-Seq data have been deposited at GEO under accession number GSE216688 and are publicly available as of the date of publication. Any additional information required to reanalyse the data reported in this paper is available from the lead contact upon request.

## Abbreviations:

ADA: adalimumab
ATAC-seq: Assay for Transposase-Accessible Chromatin using sequencing
DA: differentially accessible
DE: differentially expressed
IMIDs: Immune-mediated inflammatory diseases
OCR: open chromatin region
PBMC: peripheral blood mononuclear cells
PCA: principal component analysis
RA: rheumatoid arthritis
TNF: tumour necrosis factor
TF: transcription factors

## Permission to reproduce

The graphical abstract was created with BioRender.com
