# Supplementary material for: Identification of a transcription factor network regulating anti-TNF mediated IL10 expression in human CD4+ T cells
Source: Discov Immunol. 2024 Jul 27;3(1):kyae013. doi: 10.1093/discim/kyae013 (PMC11407445; doi:10.1093/discim/kyae013)
Supplement: kyae013_suppl_Supplementary_Figures_S1-S4 [file kyae013_suppl_supplementary_figures_s1-s4.pdf]

Fig S1

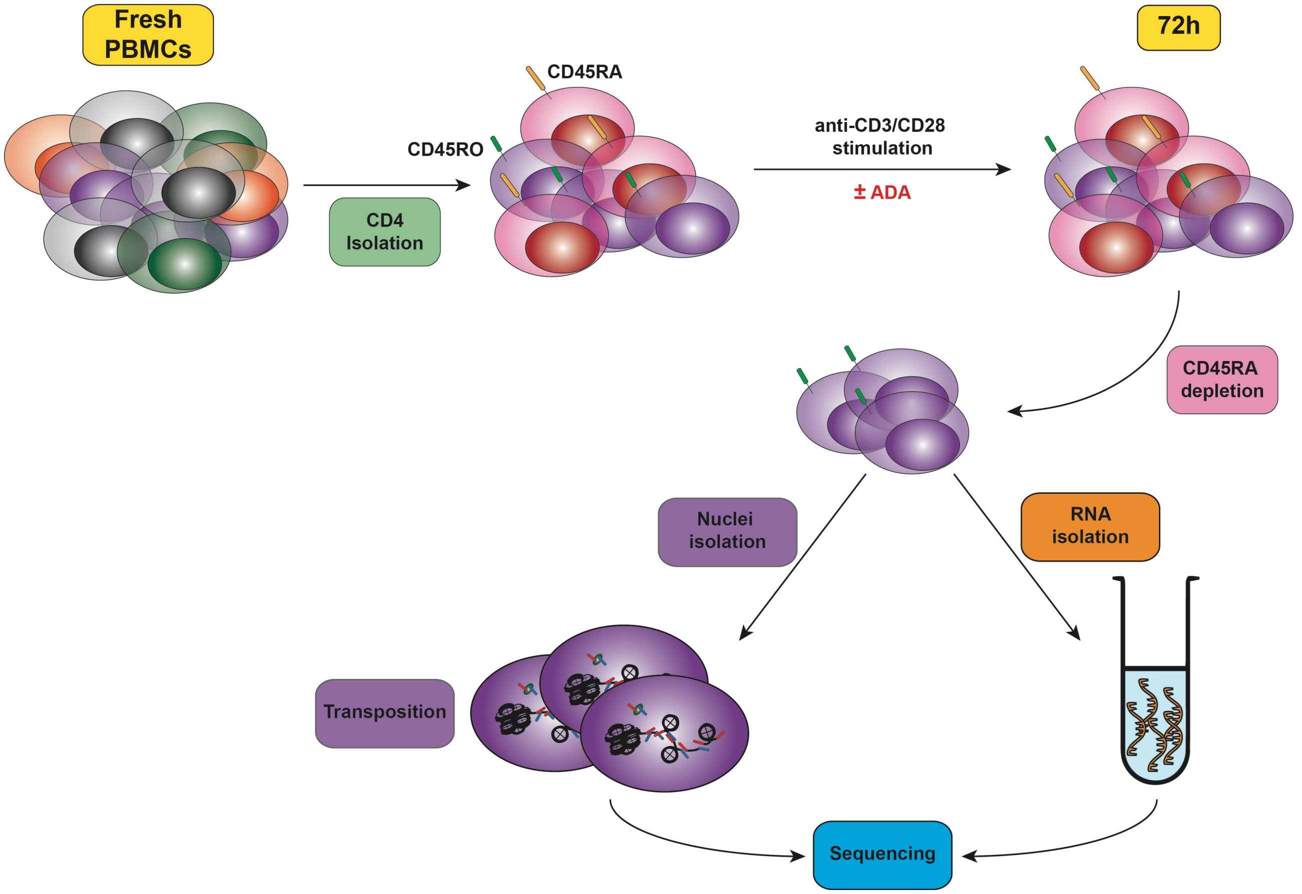

Supplementary Figure 1. Representative plot of the experimental design.

Fig S2

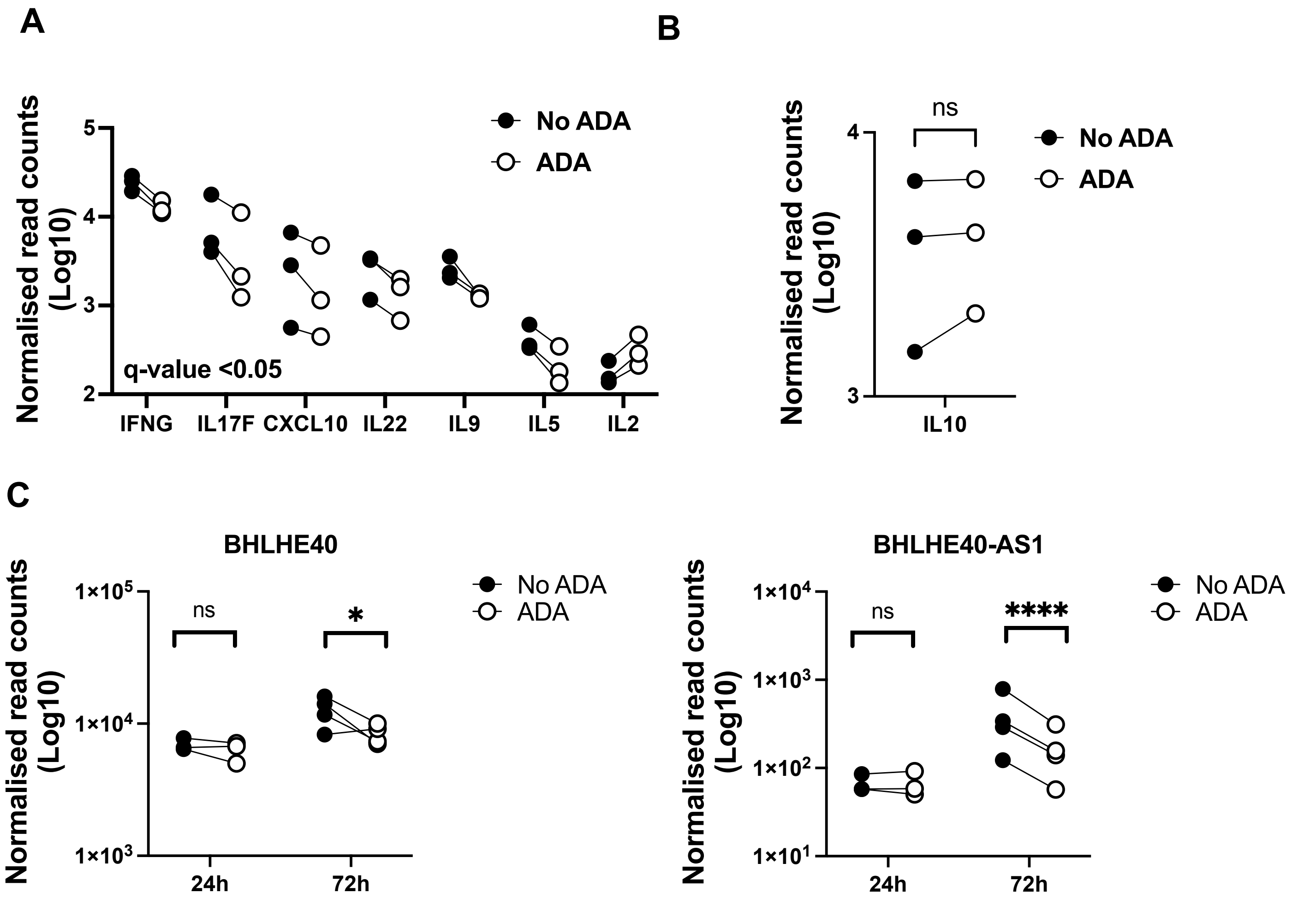

Supplementary Figure 2. Cumulative plot showing normalised read counts of DE inflammatory cytokine and chemokine (A) and anti-inflammatory IL10 (B) genes at 24h post stimulation with aCD3/CD28 mAb, in the absence (filled symbol) or presence (open symbol) of ADA. (C) Cumulative plot showing normalised read counts of *BHLHE40* (left) and *BHLHE40-AS1* (right) genes at 24h and 72h post stimulation with aCD3/CD28 mAb, in the absence (filled symbol) or presence (open symbol) of ADA; * q-value < 0.05 and **** q-value <0.0001.

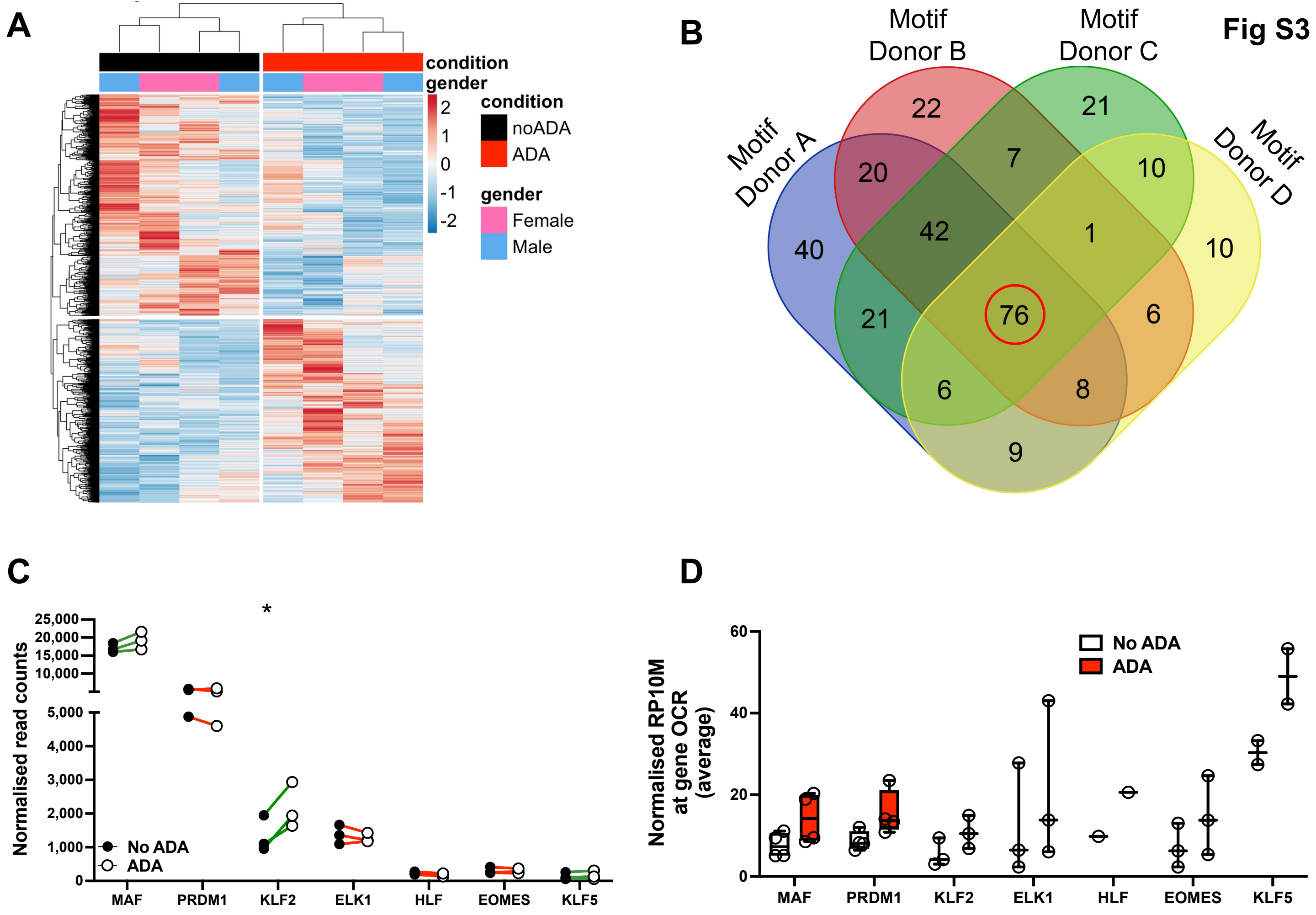

Supplementary Figure 3. (A) Heatmap showing signal intensity of differentially accessible peaks from ATAC-seq of CD4+CD45RA- T cells 72h post stimulation with aCD3/CD28 mAb in the absence vs. presence of ADA. (B) Venn diagram showing the donor overlap of TFs motifs significantly enriched ($p<0.05$) at differentially accessible (DA) OCRs in ADA treated cells at 72h post stimulation. (C) Cumulative plot showing normalised read counts of highlighted genes in Figure 3D at 24h post stimulation with aCD3/CD28 mAb, in the absence (filled symbol) or presence (open symbol) of ADA. (D) Cumulative plot showing from each donor (round symbols) averaged normalised peak intensities (“tags”) per 10 million reads (RP10M) for differential OCRs at the gene loci from Figure 3D 24h post stimulation with aCD3/CD28 mAb, in absence (white) or presence (red) of ADA.

Fig S4

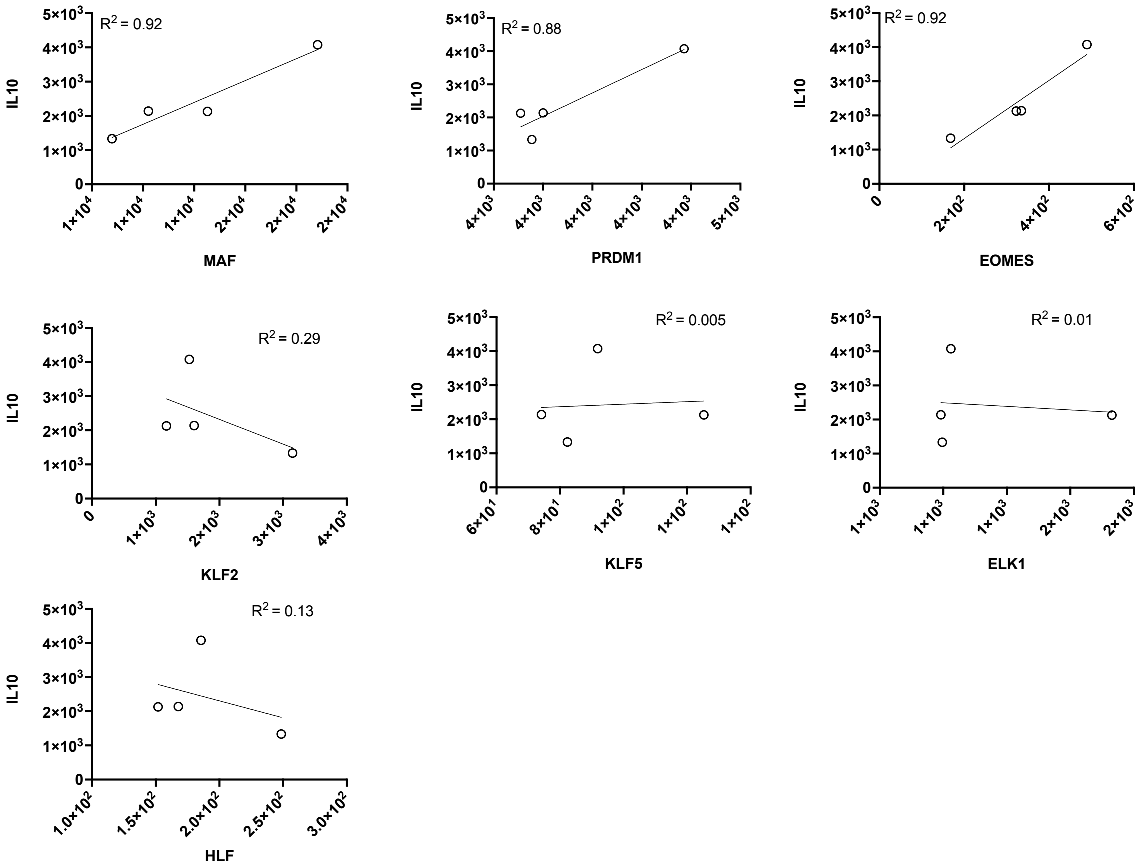

Supplementary Figure 4. Linear regression model comparing gene expression levels of IL10 with TFs from Figure 2E at 72h post treatment with anti-TNF; coefficients of determinations (r^2) are indicated**.**
